# Challenges and Opportunities in Lentivirus Viral Vector Manufacturing for In Vivo Applications

**DOI:** 10.3390/biomedicines14020369

**Published:** 2026-02-05

**Authors:** Eduardo Barbieri, Caryn L. Heldt

**Affiliations:** 1 Health Research Institute, Michigan Technological University, Houghton, MI 49931, USA; heldt@mtu.edu; 2 Department of Chemical Engineering, Michigan Technological University, Houghton, MI 49931, USA

**Keywords:** lentivirus viral vectors, in vivo cell therapy, biomanufacturing, upstream, downstream, analytical assays

## Abstract

The clinical success of chimeric antigen receptor (CAR) T-cell therapies has revolutionized oncology, yet the high costs and logistical complexities of ex vivo manufacturing remain significant barriers to global patient access. In vivo cell therapy, which involves the direct injection of lentiviral vectors (LVVs) to engineer cells within the patient’s body, offers a promising, cost-effective alternative. However, transitioning from ex vivo to in vivo applications necessitates a fundamental shift in LVV biomanufacturing to ensure safety and efficacy. This paper examines the critical bottlenecks in the current LVV production landscape. In upstream processing, we explore LVV particle assembly and maturation mechanisms, the effect of transgene size on LVV functional titers and the formation of non-functional byproducts, including empty and partially formed LVV particles and extracellular vesicles (EVs). These impurities pose severe risks of immunotoxicity and insertional mutagenesis when delivered in vivo. In downstream processing, we highlight the challenges of purifying labile LVV particles, emphasizing the need for rapid, high-resolution separation techniques like continuous processing to maintain functional titers. Furthermore, we address the limitations of current analytical assays, which often fail to distinguish mature, functional LVVs from structurally similar but inactive contaminants. We conclude that the future of in vivo lentiviral therapy depends on developing novel purification strategies based on subtle biophysical differences—such as surface charge and capsid morphology—and implementing robust, high-throughput analytics to ensure delivery of high-purity, potent therapeutic viral vectors.

## 1. Introduction

Since 2010, the treatment of cancer has been revolutionized by our ability to transduce patients’ T-cells with a chimeric antigen receptor (CAR), generating CAR-T cells. These cells are injected back into the patient after external transduction by a lentivirus vector (LVV), followed by the patient’s own T-cells targeting the cancer cells. Currently, six different cell therapies utilizing lentivirus have been approved by the FDA [1,2]. Despite the clinical success of ex vivo cell therapies, the high cost associated with these treatments represents a major challenge in democratizing access for patients globally. LVVs are responsible for 66% of the total cost associated with production materials [3]. Therefore, in vivo cell therapy has emerged as a potentially more affordable approach to engineer T-cells via direct injection of LVV particles into patients [4].

Both in vivo and ex vivo lentivirus-based cell therapies present challenges and opportunities in therapeutic applications. Besides cost, ex vivo cell therapies rely on cumbersome processes involving cell harvesting, cell culture, transduction with LVVs, and reinfusion. The complexity of these processes limits the number of available facilities with appropriate infrastructure and staff, creating logistical challenges. On the other hand, ex vivo cell therapies allow cell populations to be separated. Specific cell types can then be transduced and rigorously characterized before reinfusion, which reduces risks to patients [5]. In vivo cell therapies are less labor intensive by eliminating cell processing steps. This approach is also less invasive and potentially faster than ex vivo cell therapies. Moreover, direct injection of LVVs into a patient’s body can expand clinical applications for different tissues, including non-dividing cells, and even whole organs [6]. While this represents a significant advantage, it simultaneously poses a risk of off-target transduction, which can lead to adverse effects such as insertional mutagenesis. Other disadvantages of in vivo cell therapies include potential immune responses and the difficulty of analyzing patients’ cells to quantify transduction efficiency. While there are no FDA-approved LVV therapeutics for in vivo therapeutics, Table 1 shows some examples of clinical trials that are currently being conducted.

Lentiviral vectors (LVVs) are enveloped, single-stranded RNA viruses, measuring 80–120 nm in diameter, and derived from the human immunodeficiency virus (HIV) [8,9]. Unlike HIV, LVVs are replication-incompetent; they are engineered to lack the *env*, *vif*, and *vpr* genes responsible for pathogenicity and replication [10]. Beyond their safety profile, the ability of LVVs to stably transduce both dividing and non-dividing cells has made them the vector of choice for cell therapies [11]. In the field of cell and gene therapy, LVVs differ from more traditional viral vectors, such as adeno-associated viruses (AAVs), in that they integrate into the host cell genome. While this characteristic promotes stable transduction, it also raises concerns regarding potential random insertions and mutagenesis, which could disrupt essential genes and potentially lead to oncogenesis [12].

LVVs can be engineered to display various glycoproteins on their envelopes that aid in tropism and stability. The vesicular stomatitis virus G glycoprotein (VSV-G) pseudotype is the most widely used for ex vivo CAR-T cell therapy. However, while VSV-G LVVs are the gold standard for ex vivo applications, low stability in human serum significantly limits their in vivo utility [13]. As an alternative, the Cocal pseudotype lentivirus exhibits high stability in human serum and is currently being explored by companies such as Umoja Biopharma for in vivo applications [14,15]. Both VSV-G and Cocal glycoproteins utilize the low-density lipoprotein receptor (LDLR) for viral entry. Because LDLR is ubiquitously expressed across a wide range of mammalian tissues, these pseudotype vectors exhibit a broad tropism. Other LVV pseudotypes that are more tissue- or cell-type specific include feline endogenous retrovirus (RD114), baboon endogenous retrovirus (BaEV), rabies virus glycoprotein (RV-G), and measles [16,17,18,19].

Although in vivo LVV cell therapy will likely reduce costs to patients, current limitations must be addressed to ensure product safety. While issues of LVV sequencing, formulation, and gene integration need to be addressed, these topics are out of the scope of this review. Here, we discuss current technologies and bottlenecks in the manufacturing and analytical characterization of LVVs for in vivo applications.

## 2. LVV Upstream Production

**Figure 1:**
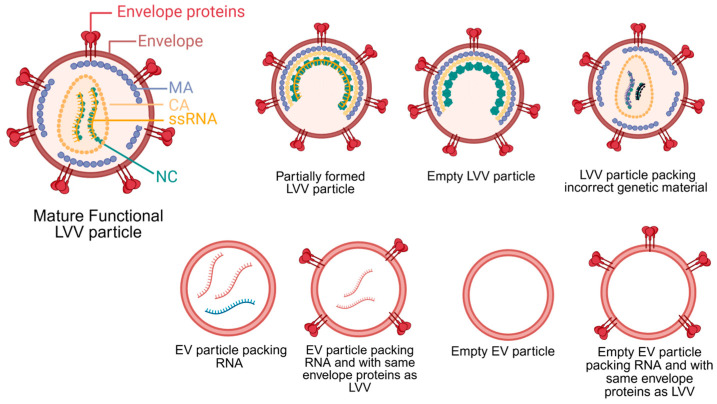
Lentivirus viral vector (LVV) structure and other potential byproducts generated during upstream production, including extracellular vesicles (EVs). During maturation, the Gag polyprotein primary structural domains, matrix (MA), capsid (CA), and nucleocapsid (NC), are cleaved by the viral protease. Only mature, functional particles can be used in in vivo applications. LVVs pack single-stranded RNA (ssRNA). Created in BioRender. Barbieri, E. (2026) https://BioRender.com/8286b5y (accessed on 27 December 2025).

LVV is produced via transient transfection of plasmids into HEK293 cells or the induction of stable cell lines. Independent of the production strategy, the primary objective of upstream processing is to maximize the yield of mature, functional LVV particles while minimizing the production of impurities. Typical impurities generated during LVV production include host cell DNA and host proteins, extracellular vesicles (EVs), and empty or partially formed LVV particles. These impurities may not pose a significant issue for ex vivo cell therapies, but they represent a substantial safety risk for in vivo applications. Figure 1 illustrates the structure of a mature LVV alongside other potential impurities with similar structure and composition. In ex vivo cell therapy, specific groups of cells are isolated, transduced and analyzed before reinjection. These steps minimize the risk of generating an immune response and potential toxicity of impurities present in the LVV samples. On the other hand, the presence of impurities in LVV for in vivo cell therapies may lead to genotoxicity, immunotoxicity, thrombogenicity, and bioaccumulation in the liver [20,21]. Therefore, minimizing production of impurities is essential to reduce potential safety risks for in vivo cell therapy using LVVs.

The stochastic nature of LVV assembly leads to LVV byproducts. During LVV particle assembly, genome packaging is driven by specific interactions between Gag polyproteins and the unspliced RNA genome [22]. Despite the high specificity between Gag and the LVV genome, studies on lentiviruses and other retroviruses have demonstrated the production of viral particles containing host cell RNA or no RNA at all [23,24,25]. Following assembly, LVV particles bud from the host cell membrane; these immature particles then undergo a maturation step mediated by the viral protease-driven cleavage of the Gag polyprotein [26]. Failure of this maturation step results in inactive, partially formed LVV particles. Although specific values for empty LVV particles are sparse in the literature, cryo-electron tomography analysis of HIV particles revealed a composition of 76% mature particles, 3% empty particles, and 21% partially formed particles [27]. Assuming similar ratios apply to LVV, the proportion of empty and partially formed particles is lower than that of other viral vectors, such as adeno-associated viruses (AAV) [28]. Nevertheless, these impurities must be removed, as they lack therapeutic efficacy, can trigger an immune response, and cause mutagenesis. Special attention must be given to particles packing wrong RNA sequences or only part of the therapeutic transgene. The injection of these particles into patients could generate altered gene sequences that could potentially lead to oncologic complications.

The engineering of different LVV pseudotypes is being investigated for in vivo applications to maximize cell- or tissue-specific transduction. One approach involves engineering the LVV envelope to display an antibody specific to a cell-type surface antigen and a fusogenic protein to maintain the LVV’s ability to trigger pH-dependent membrane fusion. This approach was successfully applied to the design of anti-CD20-targeted LVVs for the specific transduction of B cells in mouse models [29]. Similarly, the company Umoja Biopharma engineered LVV envelopes to display Cocal virus G and anti-CD3 single-chain variable fragment (scFv) proteins for the transduction of T cells in in vivo models. [15]. In another application, researchers modified the envelope glycoproteins of Tupaia paramyxovirus (TPMV) to specifically target lung cancer cells expressing IL-13 receptor alpha 2 (IL-13Rα2). Animal studies demonstrated a more than 15-fold increase in tumor transduction in mice when compared with non-engineered LVVs [30]. Although the engineering of LVV pseudotypes with higher cell and tissue specificity is essential to reduce risks to patients for in vivo applications, it also creates upstream challenges. For example, the measles virus glycoprotein pseudotype LVV showed a 2-fold reduction in functional titer when compared to VSV-G LVV [31]. Reduction in LVV functional titers increases manufacturing costs, potentially making novel therapeutics financially unviable or restricted to very few patients.

Stability in human serum is also an important attribute of in vivo LLV. Stability is typically dominated by the LVV envelope protein compositions. One study demonstrated that LVV particles pseudotyped with various envelope proteins—including murine leukemia virus (MLV), gibbon ape leukemia virus (GALV), the feline endogenous RD114 retrovirus, Moloney MLV 4070A, rabies virus glycoprotein, and measles virus—all exhibited shorter half-lives in in vitro assays at 37 °C compared to VSV-G [32]. Therefore, the engineering of novel LVV pseudotypes for in vivo applications must also assess the impact of envelope proteins on functional titers and stability.

Traditionally, proteins and viral vectors are produced by the transient transfection of plasmids into cells. However, this approach is expensive, with GMP-grade plasmids representing 36% to 46% of the total raw material cost for LVV production [33]. The use of stable cell lines offers a less expensive alternative, as they greatly reduce or eliminate the use of plasmids [34,35,36]. While monoclonal antibody (mAb) production has standardized the use of stable producer cell lines prior to Phase I clinical trials to ensure product consistency and high titers, LVV manufacturing remains largely reliant on transient transfection during early clinical stages [37]. This discrepancy is primarily driven by the inherent cytotoxicity of LVV components—such as the viral protease and specific envelope proteins—which complicates the establishment of constitutive stable lines [35,38]. Recent progress has shown that stable producer cell lines for the production of Cocal-pseudotyped LVVs show higher functional titers and lower cell toxicity when compared with the VSV-G pseudotype [39]. As the field of in vivo LVV therapies is still in the early stages, scientists have the opportunity to engineer and select LVV envelope proteins based not only on clinical outcomes but also on their feasibility for production by stable cell lines. Besides cost reduction, stable cell lines will eliminate the use of transfection reagents, therefore facilitating easier manufacturing scale-up and reducing lot-to-lot variability.

Functional LVV titers decrease as the size of the transgene to be packaged increases. The packing limit of LVV is typically considered to be 9.8–10 kb [40,41,42]. This size limit was likely derived from the wild-type genome size of HIV, which has a length of 9.8 kb [43]. However, this perceived size limit has been challenged; Kumar et al. successfully produced and quantified functional LVV particles using an 18 kb transgene [44]. The same author showed that LVV functional titers reduce as the transgene size increases even when their sizes are below 10 kb. For example, when LVV was produced with transgenes composed of only green fluorescence protein (GFP) or GFP plus Cas9 with sizes around 4.5 kb and 9.7 kb, respectively, more than one log reduction in LVV functional titer was observed for LVV packing GFP plus Cas9 as compared to the smaller GFP alone [45]. Two factors likely contributed to the effect of transgene size on LVV functional titer. First, it may be harder for the Gag polyprotein to transport larger proviral RNA from the cellular nucleus to the cytoplasm [44]. Second, a longer proviral RNA may be more difficult to assemble into a capsid by the Gag polyprotein. During viral particle assembly, RNA is packaged by direct interaction of Gag polyprotein with the RNA packing sequence [46]. Therefore, we hypothesize that an increase in transgene size makes it more difficult for the Gag polyprotein to identify and bind the RNA packaging sequence. Lower functional titers associated with an increase in transgene size may indicate a higher proportion of empty or partially formed particles. This poses a significant challenge for in vivo cell therapy, as these impurities present a safety risk for patients. Moreover, reduced titers could render LVV production economically unviable, thereby restricting the number of diseases that can be effectively targeted by in vivo therapies.

Other challenges during LVV production are the co-secretion of EVs and retro-transduction of HEK293 cells by LVV particles. Cells naturally produce EVs that can closely resemble LVVs in size, envelope protein composition, and RNA content (Figure 1) [47,48]. The presence of EVs may lead to patient complications similar to ones previously described for empty and partially formed LVV particles. Some conditions that increased EV secretion from mammalian cells are hypoxia, acidification, starvation, and hyperglycemia [49,50,51]. Developing production processes that minimize cellular exposure to these stressors could significantly reduce EV contamination. Another issue is retro-transduction. As mature LVV particles are generated, they can transduce the producer cell line, thereby reducing functional titers. This phenomenon has been observed with VSV-G pseudotyped LVVs, leading to functional titer losses ranging from 60% to 97% [35,52,53]. To minimize this loss, researchers found that reducing the culture pH from 7.2 to 6.8–6.7 renders VSV-G unable to bind to LDLR, thereby inhibiting retro-transduction [53]. However, VSV-G LVVs are not utilized in vivo in cell applications due to low stability in serum. Therefore, a similar strategy needs to be tested with Cocal LVV to confirm whether this pH-reduction strategy is equally effective for Cocal pseudotyped LVV.

Upstream processes for producing LVV particles for in vivo cell therapy must be designed to maximize functional titers while simultaneously reducing the production of impurities, especially non-functional LVV by-products. During production via the induction of stable cell lines or transient transfection, Design of Experiments (DoE) and more novel approaches exploring data-driven Bayesian optimization (BO) combined with machine learning (ML) are powerful statistical tools to determine the parameters and conditions required to maximize the yield of mature, functional LVV particles. Although, to the best of our knowledge, there are not any scientific publications specifically applying ML to the optimization of LVV production, insights can be gained from antibodies and adeno-associated virus research [54,55,56]. For example, ML could be used to expedite the design of LVV envelope proteins, including antibodies or their epitope, to enhance tissue specificity and improve transduction efficiency, while maintaining patient safety. Bayesian optimization, combined with ML, has demonstrated the capability to improve current biomanufacturing processes for AAV and mRNA by requiring fewer experiments than traditional DoE and helping select the most critical parameters to be optimized during process development [57,58]. Hopefully these approaches will also be applied to reduce the production of empty or partially formed LVVs, particles packaging incorrect RNA sequences, and extracellular vesicles.

Another approach to improve LVV production involves better understanding of the proviral RNA packaging mechanism and maturation process in engineered LVVs, with a particular focus on the role of Gag polyproteins. Elucidating these production mechanisms may create opportunities to engineer more efficient Gag polyproteins and investigate strategies to reduce the formation of non-functional particles. Furthermore, understanding why transgene size affects LVV functional titers may help expand the scope of in vivo cell therapy applications by allowing the use of longer RNA sequences with therapeutic value. Finally, production of mature functional LVV particles packing correct RNA sequences at high titers is essential to reduce risks to patients and reduce manufacturing costs for in vivo cell therapies.

## 3. LVV Downstream Purification

In vivo application of LVVs will require significant improvements to current downstream processes to ensure the removal of process- and product-related impurities while maintaining a high recovery of functional particles. The labile nature of LVVs requires purification processes with short resident times to minimize loss of functional titer. Another challenge is the removal of LVV byproducts, empty and partially formed LVV particles, and EVs during purification. These impurities present similar structure and envelope composition to functional LVV particles, challenging current available purification technologies. Finally, regulatory agencies typically require viral inactivation and sterile filtration of therapeutics produced in human cell lines before in vivo application. LVV size and structure create unique challenges during these steps, leading to a reduction in LVV functional titers. Therefore, unique characteristics of LVV structure and composition when compared to traditional protein-based therapeutics and other viruses, such as AAV, create major challenges during LVV downstream purification.

The primary challenge in LVV purification is the intrinsic instability of the viral particles. Many physicochemical factors, such as pH variation, temperature, shear stress, and ionic strength, can quickly reduce the LVV functional titer [32,59]. Therefore, minimizing processing time is crucial for maximizing yield. While the general trend in biomanufacturing is toward increasing productivity, many legacy processes are designed to maximize the binding capacity of chromatographic resins, thus increasing residence time. However, longer residence times significantly reduce the recovery of functional LVVs. Studies by Pamenter et al. demonstrated that extended contact between LVV particles and chromatographic substrates leads to irreversible adsorption, causing a reduction in functional recovery of over 50% [60,61]. The complexity and diverse composition of LVV envelope proteins create multipoint attachment via electrostatic and hydrophobic interactions with chromatographic surfaces, leading to irreversible structural changes in the bound state [60]. To mitigate this, the use of membranes or monoliths instead of traditional resin-based beads offers a viable alternative; these technologies operate at shorter residence times without the limitations of backpressure [62]. Another strategy to minimize processing duration is the implementation of continuous downstream processing integrated with perfusion production systems [63]. By continuously loading the LVVs produced in the bioreactor directly into a purification system, hold-up times between production and purification are minimized, thereby reducing both degradation and retro-transduction within the bioreactor. One potential downstream process that allows for a truly continuous purification train is aqueous two-phase systems (ATPS). Recoveries as high as 99% have been achieved with significantly shorter processing times than conventional chromatography [64,65]. Regardless of the specific purification strategy, minimizing processing time remains paramount to ensuring the recovery of high-quality, functional particles.

**Figure 2:**
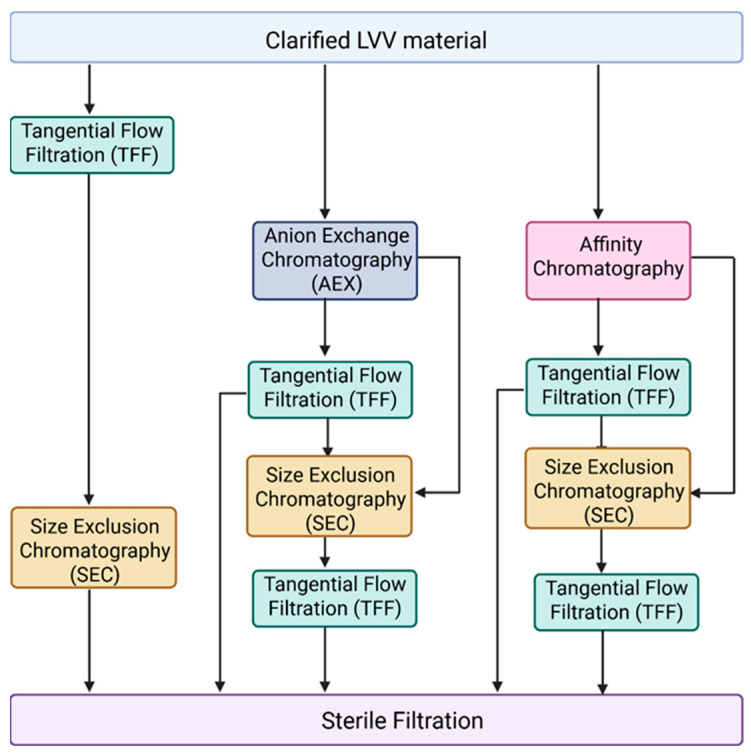
Different combinations of unit operations applied during LVV purification. Following LVV production in HEK293, cell culture fluid is clarified by centrifugation or depth filtration for removal of cells and other large particles. Anion exchange chromatography (AEX) and affinity chromatography are typically used as a capture step. During downstream tangential flow filtration (TFF), it is applied for LVV concentration and/or buffer exchange. Size exclusion chromatography (SEC) can be used as a polishing step. Finally, LVV undergoes sterile filtration.

Another major challenge in LVV purification for in vivo applications is the effective removal of empty, partially formed LVV particles and extracellular vesicles. Currently, most purification processes described in the literature are optimized for VSV-G pseudotyped LVVs intended for ex vivo applications [62,66,67]. Figure 2 illustrates the unit operations typically employed for VSV-G LVV purification, which generally include tangential flow filtration (TFF) for concentration and diafiltration, anion-exchange (AEX) or affinity chromatography for capture in bind-and-elute mode, and size-exclusion chromatography (SEC) combined with mixed-mode chromatography as a polishing step in flow-through mode. However, these established processes often fail to address the removal of empty and partially formed particles, or EVs, which share similar physical properties with functional mature LVVs. Furthermore, in vivo therapeutics necessitate the use of alternative pseudotypes, such as Cocal, for which purification data remains scarce. Therefore, we recommend a primary strategy focused on identifying subtle biophysical differences between empty LVVs, mature LVVs, and EV subpopulations—specifically regarding surface charge density and hydrophobicity—to identify suitable chromatographic strategies. Given the expected similarities between these particles, achieving high-resolution separation will likely require extensive screening of salts and buffer systems during gradient elution. A complicated factor in this screening process is the labile nature of lentiviruses; beyond merely achieving purity, it is essential to ensure that buffer composition, ionic strength, and pH do not inactivate or aggregate the LVV particles. Despite these challenges, the FDA and other regulatory agencies are expected to impose stricter purity requirements for in vivo applications to ensure patient safety.

The development of different LVV pseudotypes to improve cell and tissue specificity for in vivo applications creates new challenges in downstream processing. It will be more difficult to establish a universal purification platform, as processes will need to be individually created and optimized according to the protein composition of the LVV envelope. For example, protein L affinity chromatography could be used to purify LVV engineered with scFvs, depending on their orientation within the envelope protein. However, the low pH required for elution of scFvs from Protein L will likely inactivate LVV particles, precluding its use. Therefore, we believe that initial purification strategies for novel LVV pseudotypes will still rely on the traditional platforms, such as anion-exchange (AEX) and mixed-mode chromatography, originally developed for VSV-G LVVs. However, the potential for reduced stability in engineered in vivo vectors will require careful process optimization to ensure gentle elution conditions that minimize the loss of transduction potency. Currently, the primary manufacturing challenge remains improving recovery, as current processes often yield less than 20% of the total product [29].

Screening of buffers, salts, and other excipients is essential for the successful manufacturing and formulation of novel LVV pseudotypes developed for in vivo applications. LVV aggregation has been reported frequently during downstream processing and final formulation [68,69]. Sulfonated buffers, such as PIPES (piperazine-N,N′-bis(2-ethanesulfonic acid)), HEPES (4-(2-hydroxyethyl)-1-piperazineethanesulfonic acid), and MOPS (3-(N-morpholino)propanesulfonic acid), have been shown to improve LVV stability and reduce aggregation [66,69,70]. More recently, arginine—even at high concentrations and in combination with a phosphate buffer—has been shown to improve the colloidal stability of LVV particles, preventing aggregation and functional titer loss [71]. However, most of these studies were developed specifically for VSV-G pseudotypes and may not directly apply to novel in vivo LVV variants; indeed, research has shown that LVVs engineered with scFvs are more prone to aggregation [72]. Beyond ensuring high recovery during purification, investigating optimal buffers and salts will facilitate the development of formulations that enhance the shelf life of LVV therapeutics, guaranteeing their therapeutic value from drug substance production to patient delivery.

Viral clearance and sterile filtration are standard FDA requirements for therapeutics produced in human cell lines. However, performing viral clearance for a viral-based therapeutic presents a unique challenge. While not a direct answer to the clearance requirement, the FDA has published guidelines for the testing of replication-competent retroviruses (RCR), which specifically details testing for lentivirus-based therapeutics [73]. The agency also recommends using gammaretrovirus RCR standard stocks as a positive control. As LVV manufacturing evolves, “spiking” studies should be conducted using model viruses to validate the ability of the purification process to remove endogenous and adventitious viral contaminants. Regarding sterility, 0.2 µm terminal filtration of LVVs has been reported in the literature to result in titer losses of approximately 30–50% [74]. However, a recent study demonstrated that these losses can be reduced to 20% by utilizing hydrophilic polyvinylidene fluoride (PVDF) filters [75].

Closed, fully integrated end-to-end viral production and purification systems have emerged in recent years as a strategy to simplify unit operations and reduce costs associated with manufacturing cell and gene therapies [76,77,78]. These closed-loop systems rely heavily on process automation combined with computational models, in-line sensors, and process analytical technology (PAT), which reduces contamination risks, batch-to-batch variability, and overall costs. In 2025, uBrinGene Biosciences Inc. (Germantown, MD, USA) released the LVV Turbo™ Platform, which features a fully closed downstream purification system [79]. While specific technical details remain proprietary, the platform reportedly eliminates the need for sterile filtration, achieves an LVV recovery higher than 60%, and increases productivity five-fold. Provided that the downstream chromatography steps are pseudotype-agnostic, this technology could significantly expedite process development of LVVs for in vivo applications.

Currently, the rapid evolution of LVV applications is pushing existing downstream processes to their limits, particularly regarding the stringent purity required for in vivo use. The engineering of particles with novel envelope proteins necessitates the development of pseudotype-specific processes to ensure high recovery of functional LVV particles. Regardless of the pseudotype, downstream strategies must prioritize the removal of specific LVV byproducts. Consequently, new purification platforms are required to exploit biophysical differences—such as charge and hydrophobicity—between active mature particles and their impurities. Since the cleavage of Gag polyproteins during maturation likely induces structural changes in the LVV envelope, these shifts represent a critical lever for process development. To maintain potency, these advanced platforms must also minimize processing time. Ultimately, achieving clinical-grade purity for next-generation therapies will require a shift toward novel downstream technologies that move beyond the constraints of traditional chromatography.

## 4. Analytical Assays for LVV

LVV concentrations are expressed either as physical titers—typically measured as particle or viral genome concentration—or as functional titers in transduction units (TU/mL). Assays designed to quantify physical titers aim to determine the total number of particles by quantifying (i) the capsid protein p24 via ELISA [80]; (ii) the LVV viral genome by reverse-transcriptase quantitative polymerase chain reaction (RT-qPCR) [81]; and more recently, (iii) particle size by multi-angle light scattering (MALS) [82]; and/or (iv) a combination of light scattering with fluorescent tagging of LVVs via flow virometry (FVM) [83,84]. Functional titers, which also serve as potency assays, are determined by measuring the number of cells successfully transduced following incubation with the LVV. The method for quantifying transduced cells varies depending on the transgene packaged within the LVV and the resulting protein expression [85,86,87,88]. Potency assays will be essential in in vivo applications to ensure patients are receiving functional LVV particles.

Quantification of LVV potency titers for in vivo applications will require more than just standard in vitro cell-based assays. As previously described, LVV envelopes are being engineered with various proteins, including scFvs, to ensure the transduction of specific cell types. Therefore, the first challenge involves identifying a cell line equivalent to the cells targeted for LVV transduction in the human body. Mata-Molanes et al. (2025) developed a potency assay for CAR-encoding LVVs using Jurkat cells and patient-derived T cells [89]. Beyond traditional in vitro assays to measure cell transduction, potency testing should include assessments of serum stability and phagocytosis resistance [90,91,92,93]. Furthermore, serum stability assays must evaluate whether pre-existing antibodies in a patient could inactivate engineered LVVs. Although an expanded analytical panel will likely increase manufacturing costs, these detailed assessments will facilitate the regulatory approval process. To this end, the FDA has released guidance documents to ensure the potency of cellular and gene therapy products, encompassing lot release testing, stability evaluation, and comparability studies following manufacturing process changes [94].

Other major challenges in LVV quantification are (i) the inability of physical assays to distinguish mature, functional LVV particles from EVs or immature LVVs and (ii) the difficulty in correlating physical particle counts with functional titers. For example, the p24 ELISA measures the total amount of p24 protein in a sample but cannot distinguish between p24 assembled into a capsid and unassembled, free-floating protein. Despite this limitation, the ratio of transduction units (TU) to the total number of particles is frequently used as an indicator of the “enrichment” or specific activity of functional LVV particles during downstream processing [45,67]. However, an “ideal” ratio remains undefined, as literature values for multiplicity of infection (MOI) vary widely—from 0.5 to 100—depending on the target cell line [95,96,97]. Other physical techniques face similar constraints; inactive LVVs may still contain a viral genome (detected by qPCR) or be an assembled particle (detected by MALS or FVM).

The challenge remains in quantifying partially formed particles in the presence of mature LVVs. Current cryo-electron microscopy (cryo-EM) methods used for visualizing these differences are cost-prohibitive and lack high-throughput capability. A known structural differentiator is the capsid shape, which transitions from spherical (immature) to conical (mature) during maturation [26]. However, the lipid envelope makes it difficult for most sensors to detect the internal capsid morphology. EVs add further complexity; although they lack a capsid, they possess similar envelope structures and sizes and can even package host–cell ssRNA.

A robust panel of analytical assays will be essential to obtain approval during the regulatory approval process. Based on published FDA guidance, key requirements include a matrix of assays to ensure product potency, RCR (replication-competent retrovirus) testing, specificity to target organs or tissues, and product stability from manufacturing through patient injection [73,94,98]. Current FDA guidelines for RCR testing require 28 days: a 21-day amplification phase, in which the final LVV product is added to a permissive cell line, followed by a 7-day indicator phase, where supernatant from the first phase is added to a naive batch of cells. Materials are then analyzed for the p24 protein via ELISA and for RCR-specific DNA sequences via qPCR or ddPCR. Beyond the lengthy duration, these assays require the selection or development of permissive cell lines tailored to the engineered LVV pseudotype that can remain viable during extended in vitro culture, which is still a challenge. Furthermore, the FDA-recommended positive control (gammaretrovirus RCR) may be unsuitable for certain permissible cell lines [73]. Regarding specificity to target organs or tissues, manufacturers must demonstrate that in vivo LVV treatment results in cell-type-specific transduction. According to the FDA, special attention must be given to ensuring the vector does not cause inadvertent germline integration [94,98]. In terms of opportunities, in 2026, the FDA announced increasing flexibility on the manufacturing and control requirements to boost innovation in cell and gene therapies [99]. This suggests the agency may allow early-phase clinical trials to proceed with less restrictive product quality release criteria.

Future research must investigate unique biophysical signatures that distinguish mature LVVs, partially formed LVVs, and EVs. We believe a critical area of development will be the identification of methods to detect the presence and morphology of the internal capsid within the envelope. These methods need to be performed in under 24 h for quick release of batches. Finally, investigating the specific mechanisms of LVV inactivation will be essential to identifying the subtle differences between active and inactive particles, which could then be exploited to improve the accuracy of next-generation analytical assays.

## 5. Conclusions

The success of LVV as an in vivo therapeutic will heavily rely on improvement of current biomanufacturing processes. While as a starting point, scientists tend to use technologies already available for production, purification and characterization of proteins and other viruses, the unique structure of LVVs and its labile structure will require novel approaches. Special focus needs to be given to EVs, immature and empty LVV particles and how to minimize their production, removal during purification, and characterization. Although challenging due to their similarities to mature active LVVs, these steps will be crucial to ensure product safety. While biomanufacturing practices are not the only steps needed to ensure a safe in vivo LVV product, these steps are essential to move this life-altering therapeutic class into the clinic.

## Figures and Tables

**Table 1:** Examples of clinical trials investigating the use of in vivo LVV therapies [7].

Targeted Clinical Conditions	Clinical Phase	Sponsor	Clinical Trial ID
Adenosine deaminase severe combined immunodeficiency	Phase I/II	Shenzhen Geno-Immune Medical Institute	NCT03645460
Relapsed/refractory multiple myeloma	Phase I	Tongji Hospital	NCT06791681
Genetic disorder X-linked severe combined immunodeficiency (SCID-X1)	Phase I/II	Shenzhen Geno-Immune Medical Institute	NCT03217617
Refractory/relapsing B-cell malignancies.	Phase I	Gilead Sciences	NCT06539338
Epilepsy	Phase I/IIa	University College, London	NCT04601974
Chronic Granulomatous Disease	Phase I/II	University of California, Los Angeles	NCT02234934

## Data Availability

No new data were created or analyzed in this study.

## Abbreviations

The following abbreviations are used in this manuscript:

AAV	Adeno-associated virus
AEX	Anion-exchange chromatography
ATPS	Aqueous two-phase systems
BaEV	Baboon endogenous retrovirus
BO	Bayesian optimization
CA	Capsid (structural domain of Gag)
CAR	Chimeric antigen receptor
Cryo-EM	Cryo-electron microscopy
DoE	Design of Experiments
ELISA	Enzyme-linked immunosorbent assay
EVs	Extracellular vesicles
FDA	Food and Drug Administration
FVM	Flow virometry
GALV	Gibbon ape leukemia virus
HEK293	Human embryonic kidney 293 cells
HEPES	4-(2-hydroxyethyl)-1-piperazineethanesulfonic acid
HIV	Human immunodeficiency virus
LDLR	Low-density lipoprotein receptor
LVV	Lentiviral vector
MA	Matrix (structural domain of Gag)
MALS	Multi-angle light scattering
ML	Machine learning
MLV	Murine leukemia virus
MOI	Multiplicity of infection
MOPS	3-(N-morpholino)propanesulfonic acid
NC	Nucleocapsid (structural domain of Gag)
p24	Lentiviral capsid protein (24 kDa)
PIPES	piperazine-N,N′-bis(2-ethanesulfonic acid)
PVDF	Polyvinylidene fluoride
RCR	Replication-competent retrovirus
RD114	Feline endogenous retrovirus
RT-qPCR	Reverse-transcriptase quantitative polymerase chain reaction
RV-G	Rabies virus glycoprotein
SEC	Size-exclusion chromatography
scFv	single-chain variable fragment
ssRNA	Single-stranded RNA
TFF	Tangential flow filtration
TU	Transduction units
VSV-G	Vesicular stomatitis virus G glycoprotein
